# Genomic spectrum of actionable alterations in serial cell free DNA (cfDNA) analysis of patients with metastatic breast cancer

**DOI:** 10.1038/s41523-024-00633-7

**Published:** 2024-04-11

**Authors:** Yael Bar, Jennifer C. Keenan, Andrzej Niemierko, Arielle J. Medford, Steven J. Isakoff, Leif W. Ellisen, Aditya Bardia, Neelima Vidula

**Affiliations:** 1 Massachusetts General Hospital Cancer Center, Boston, MA USA; 2 Tel Aviv Sourasky Medical Center and The Faculty of Medicine, Tel Aviv University, Tel Aviv, Israel; 3 Harvard Medical School, Boston, MA USA

**Keywords:** Breast cancer, Genetics research

## Abstract

We aimed to study the incidence and genomic spectrum of actionable alterations (AA) detected in serial cfDNA collections from patients with metastatic breast cancer (MBC). Patients with MBC who underwent plasma-based cfDNA testing (Guardant360^®^) between 2015 and 2021 at an academic institution were included. For patients with serial draws, new pathogenic alterations in each draw were classified as actionable alterations (AA) if they met ESCAT I or II criteria of the ESMO Scale for Clinical Actionability of Molecular Targets (ESCAT). A total of 344 patients with hormone receptor-positive (HR+)/HER2-negative (HER2-) MBC, 95 patients with triple-negative (TN) MBC and 42 patients with HER2-positive (HER2 + ) MBC had a baseline (BL) cfDNA draw. Of these, 139 HR+/HER2-, 33 TN and 13 HER2+ patients underwent subsequent cfDNA draws. In the HR+/HER2- cohort, the proportion of patients with new AA decreased from 63% at BL to 27–33% in the 2nd-4th draws (*p* < 0.0001). While some of the new AA in subsequent draws from patients with HR+/HER2- MBC were new actionable variants in the same genes that were known to be altered in previous draws, 10-24% of patients had new AA in previously unaltered genes. The incidence of new AA also decreased with subsequent draws in the TN and HER2+ cohorts (TN: 25% to 0–9%, HER2 + : 38% to 14–15%). While the incidence of new AA in serial cfDNA decreased with subsequent draws across all MBC subtypes, new alterations with a potential impact on treatment selection continued to emerge, particularly for patients with HR+/HER2- MBC.

## Introduction

The field of precision oncology in which clinical decision making is based on a patient’s specific tumor genomic profile rather than a one-size-fits-all treatment algorithm, has rapidly evolved over the past decade. This approach has improved outcomes in several cancer types including breast cancer, mainly in the metastatic setting^1,2^. As a result, screening for actionable genomic alterations (AA, also known as targetable alterations), which enables the use of genotype-matched therapies, is increasingly performed for patients with metastatic breast cancer (MBC). Following the approval of the PI3K inhibitor alpelisib for patients with *PIK3CA*-mutated hormone receptor-positive/HER2-negative (HR+/HER2-) MBC^3^ and the oral selective estrogen receptor degrader (SERD) elacestrant for patients with *ERS1*-mutated HR+/HER2- MBC^4^, genomic tests are now commonly employed in HR+/HER2- MBC to identify *PIK3CA* or *ESR1* mutations.

The ESMO Scale for Clinical Actionability and Molecular Targets (ESCAT), first published in 2018, is a framework to aid in the categorization of genomic alterations according to the level of evidence (LOA) available for alteration-drug match^5–7^. Alterations are ranked as ESCAT I if alteration-drug match was associated with improved outcomes in clinical trials, and ESCAT II if alteration-drug match was associated with antitumor activity in prior clinical studies. ESCAT III/IV categories define alterations with lower LOA^5^. The pivotal SAFIR02-BREAST study evaluated the clinical utility of using tumor-specific genomic alterations to guide treatment recommendations in patients with MBC. The study showed improved outcomes with genotype-matched therapies compared to chemotherapy only in the setting of ESCAT I/II alterations with no benefit in ESCAT III/IV altered tumors^8^.

Blood-based biopsy (liquid biopsy) is a non-invasive method for the detection of tumor-specific genomic alterations through the analysis of cell-free DNA (cfDNA) shed into the blood by the tumor. The relatively simple and non-invasive nature of cfDNA analysis makes it an effective tool for monitoring tumor heterogeneity and tracking clonal evolution over time^9,10^. Thus, serial cfDNA analysis throughout a patient’s disease course is increasingly used to guide clinical treatment decisions for patients with MBC^11–13^. However, the utility of serial cfDNA testing in identifying new AA that may affect treatment selection is not well understood.

We aimed to evaluate the genomic spectrum of AA (defined as ESCAT I/II alterations) in serial cfDNA analysis of patients with MBC, across disease subtypes. Specifically, we report the incidence of newly emerged AA in serial cfDNA testing, the frequency of new AA by altered genes, and the correlation between prior treatments received and the incidence of new AA.

## Results

### Patients

**Fig. 1:**
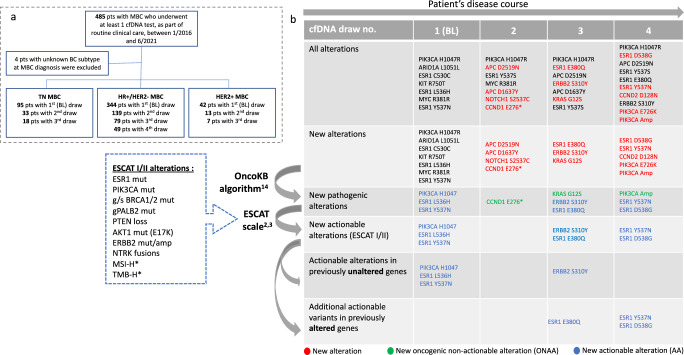
Study design. **a** Study cohort diagram. **b** Serial cfDNA results for an individual representative patient with HR+/HER2− MBC. Columns indicate serial cfDNA draws. Rows indicate the steps taken to analyze cfDNA results for each patient. * Data on alteration was not available for this analysis. pts patients, MBC metastatic breast cancer, BL baseline, g/s germline/somatic, mut mutant, amp amplification.

Four hundred and eighty-five patients with MBC underwent at least one cfDNA test (Guardant360®) between 1/2016 and 6/2021. Four patients with unknown MBC subtype were excluded. Of the remaining 481 patients, 344 had HR+/HER2- MBC, 95 had TN MBC, and 42 had HER2 + MBC. Figure 1a summarizes the number of patients in each cohort who underwent subsequent serial cfDNA draws during their disease course. Table 1 summarizes the demographics of patients who underwent serial cfDNA draws as well as the timing and type of draws.

**Table 1:** Characteristics of patients undergoing cfDNA testing

cfDNA draw no.	1 (BL)	2	3	4
HR+/HER2- MBC, *n*	344	139	79	49
Median Age at draw (range)	60 (30–87)	60 (30–88)	59 (37–89)	58 (38–82)
Median No. of prior therapies (range)	1 (0–14)	3 (0–12)	3 (1–13)	4 (1–11)
Mean time interval from prior draw, months (range)	N/A	7.8 (1–43)	6.3 (1–34)	6.4 (1–29)
Draw type, *n* (%)^a^		N/A	N/A	N/A
Pre-treatment	99 (29)	85 (61)	64 (81)	41 (84)
Post-treatment discontinuation	204 (59)	54 (39)	15 (19)	8 (16)
On-treatment	41 (12)	–	–	–
Prior therapies, *n* (%)^b^				
AI	269 (78)	120 (86)	73 (92)	46 (96)
SERD	134 (39)	96 (69)	60 (76)	39 (81)
CDK4/6 inhibitor	161 (47)	106 (76)	59 (75)	44 (92)
PIK3 inhibitor	32 (9)	18 (13)	8 (10)	8 (17)
Chemotherapy	113 (33)	55 (40)	38 (48)	29 (60)

TN MBC, *n*	95	33	18	
Median Age at draw (range)	56 (30–91)	56 (31–92)	56 (34–71)	
Median No. of prior therapies (range)	1 (0–8)	2 (1–9)	3 (1–9)	
Mean time interval from prior draw, months (range)	N/A	5.4 (1–18)	5.1 (1–22)	
Draw type, *n* (%)^a^				
Pre-treatment	35 (37)	N/A	N/A	
Post-treatment discontinuation	56 (59)	22 (67)	15 (83)	
On-treatment	4 (4)	11 (33)	3 (17)	
Prior therapies, *n* (%)^b^				
Chemotherapy	53 (56)	23 (70)	12 (67)	
Immunotherapy	13 (14)	8 (24)	6 (33)	
ADC	7 (7)	10 (30)	11 (61)	

HER2 + MBC, *n*	42	13	7	
Median Age at draw (range)	56 (30–78)	54 (32–71)	60 (39–72)	
Median No. of prior therapies (range)	1 (0–9)	2 (1–7)	2 (1–6)	
Mean time interval from prior draw, months (range)	N/A	7 (1–25)	6 (1–16)	
Draw type, *n* (%)^a^				
Pre-treatment	17 (40)	N/A	N/A	
Post-treatment discontinuation	19 (45)	6 (46)	4 (57)	
On-treatment	6 (14)	7 (54)	3 (43)	
Prior therapies, *n* (%)^b^				
Chemotherapy	14 (33)	11 (85)	6 (86)	
Anti-HER2 therapy (including HER2 targeted ADCs)	21 (50)	12 (92)	7 (100)	

*AI* aromatase inhibitor, *SERD* selective estrogen receptor degrader, *ADC* antibody-drug conjugate.

^a^Pre-treatment, no therapies were given prior to cfDNA draw (draws were done at MBC diagnosis) or cfDNA draw occurred <30 days after the start of the 1st line of therapy for MBC; on-treatment, cfDNA draw occurred >30 days after starting therapy and <30 days before ending therapy; post-treatment discontinuation, cfDNA draw was taken <30 days before ending therapy and <30 days after starting the next therapy. The vast majority of post-treatment discontinuation draws occurred at time of progression.

^b^Therapies were given for metastatic disease only, except for AI which may have been received in the adjuvant and/or the metastatic setting. The proportions of prior therapies received do not sum to 100% as patients could have received more than one prior therapy.

In the HR+/HER2- cohort the mean time interval between draws ranged between 6.3 to 7.4 months. The median number of prior lines of therapies was 1 prior to the BL draw, 3 prior to the 2nd and the 3rd draws, and 4 prior to the 4th draw. For 29% of patients, the BL draw was taken upon MBC diagnosis prior to any therapy (pre-treatment). However, most of the draws were taken upon treatment progression (post-treatment discontinuation) regardless of draw number (59%, 61%, 81%, and 84% of patients in the BL, 2nd, 3rd, and 4th draw groups, respectively). As expected, exposure to prior treatments is increased as the number of prior draws increases. For example, 76% of patients were treated with an aromatase inhibitor (AI) in the adjuvant or the metastatic setting prior to the BL draw, while 96% of patients received AI prior to the 4th draw. Additional treatments given prior to draws are listed in Table 1.

In the TN cohort, the median number of therapies before draws were 1 prior to the BL draw, 2 prior to the 2nd, and 3 prior to the 3rd draw. The mean time interval between draws was shorter than in the HR+/HER2- cohort and ranged from 5.1 to 5.4 months. Thirty-seven percent of the BL draws were taken upon MBC diagnosis but similarly to the HR+/HER2-cohort, the majority of the draws were taken upon treatment progression (post-treatment discontinuation). 56% of patients were exposed to chemotherapy prior to the BL draw but only a minority were exposed to immunotherapy or antibody-drug conjugates (ADCs) (14% and 7%, respectively).

In the HER2+ cohort, the median number of therapies before draws was 1 prior to the BL draw, and 2 prior to the 2nd and 3rd draws. The mean time interval between draws was 6–7 months. 40% of the BL draws were taken upon MBC diagnosis. While only 33% and 50% of patients received chemotherapy and anti-HER2 therapy prior to the BL draw, respectively, the majority of patients were exposed to these treatments prior to the 3rd draw (85% and 92%, respectively).

### Spectrum of new genomic alterations in serial cfDNA draws

**Fig. 2:**
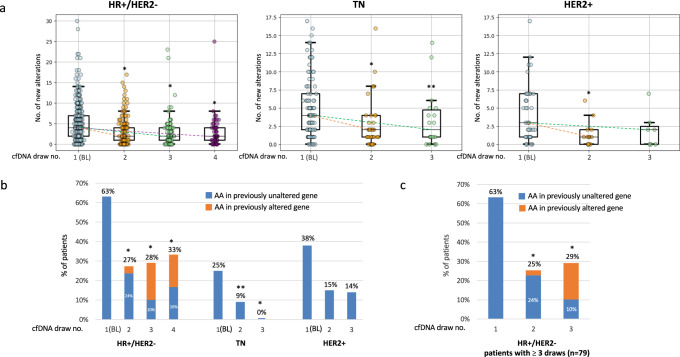
New genomic alterations identified in serial cfDNA analysis. **a** Box plots show total number of new alterations (actionable and non-actionable). Each point represents a patient and lines within boxes indicate the median number of new alterations. The boxes span the inter-quartile range (IQR). Whiskers span 1st quartile −1.5 × IQR and 3rd quartile +1.5 IQR; and points above and below whiskers represent outliers. Dashed lines between boxes represent the trends in the median number of new alterations between the BL draw and each of the subsequent draws. **b** Incidence of new AA. **c** incidence of new AA in patients with ≥3 draws in the HR+/HER2- cohort. *Significant decrease compared to BL draw (*p* < 0.05). **p = 0.05. *In the BL draw all alterations were considered new*.

The median number of alterations was generally higher in subsequent draws than in BL in the HR+/HER2- and TN cohorts, but was not significantly changed in the HER2+ cohort (Table 2). However, the median number of new genomic alterations significantly decreased in all subtypes with subsequent draws (Table 2, Fig. 2a, and Supplementary Fig. 1). The overall landscape of pathogenic alterations (actionable and non-actionable) in the BL draw according to subtype is presented in Supplementary Fig. 2.

**Table 2:** Characteristics of new genomic alterations identified in serial cfDNA analysis

cfDNA draw no.	1 (BL)^a^	2		3		4	
HR+/HER2- MBC, *n*	344	139	*p* (vs. BL)	79	*p* (vs. BL)	49	*p* (vs. BL)
Median no. of alterations	4	5	0.09	5	0.01	6	0.006
Median no. of new alterations	4	2	<0.0001	2	<0.0001	2	0.0009
% Of Pt. w/ new AA (ESCAT I/II) (95% CI)	63% (58–68%)	27% (20–35%)	<0.0001	28% (19–39%)	<0.0001	33% (20–47%)	<0.0001
% of Pt. w/ new AA (ESCAT I/II) in previously unaltered genes (95% CI)	N/A	24% (17–32%)	N/A	10% (4–19%)	N/A	16% (7–30%)	N/A
% Of Pt. w/ new ONAA (95% CI)	69% (64–74%)	47% (39–56%)	<0.0001	51% (39–62%)	<0.0001	61% (46–75%)	<0.0001
% Of Pt. w/ no new AA or ONAA (95% CI)	17% (14–22%)	43% (35–52%)	<0.0001	38% (27–50%)	<0.0001	29% (17–43%)	0.06

TN MBC, *n*	95	33	*p* (vs. BL)	18	*p* (vs. BL)		
Median no. of alterations	4	5	0.62	5	0.14		
Median no. of new alterations	4	2	0.0009	2	0.05		
% Of Pt. w/ new AA (ESCAT I/II) (95% CI)	25% (17–35%)	9% (2–24%)	0.05	0% (0–0)	0.02		
% Of Pt. w/ new ONAA (95% CI)	84% (75–91%)	58% (39–74%)	<0.0001	50% (26–74%)	<0.0001		
% Of Pt. w/ no new AA or ONAA (95% CI)	12% (6–20%)	42% (25–61%)	0.0001	50% (26–74%)	<0.0001		

HER2+MBC, *n*	42	13	*p* (vs. BL)	7	*p* (vs. BL)		
Median no. of alterations	3	2	0.13	3	0.63		
Median no. of new alterations	3	1	0.005	2	0.12		
% Of Pt. w/ new AA^b^(ESCAT I/II) (95% CI)	38% (23–54%)	15% (2–44%)	0.13	14% (0.4–58%)	0.22		
% Of Pt. w/ new ONAA (95% CI)	69% (53–82%)	54% (25–80%)	0.02	43% (10–82%)	0.22		
% Of Pt. w/ no new AA or ONAA (95% CI)	21% (10–37%)	46% (19–75%)	0.08	43% (10–82%)	0.22		

*AA* actionable alteration, *ONAA* oncogenic non-actionable alteration, *ESCAT* ESMO scale for clinical actionability of molecular targets.

^a^In the BL draw all alterations were considered new.

^b^ERBB2 amplification was not considered as new AA for patients with HER2 + MBC.

In the HR+/HER2- cohort, the proportion of patients with new AA (ESCAT I/II) significantly decreased in subsequent draws compared to the BL draw. While 63% of patients had AA in their BL draw, 27–33% had new AA in subsequent draws. While most of the patients with new AA in the 2nd draw had new AA in previously unaltered genes (24% out of 27%), only the minority of patients with new AA in the 3rd and the 4th draws had new AA in previously unaltered genes (10% out of 28%, and 16% out of 33%, respectively) (Table 2 and Fig. 2b) and the majority had new actionable variants in previously altered genes. Additionally, the incidence of new oncogenic non-actionable alterations (ONAA) significantly decreased with subsequent draws. Complementary to this, subsequent draws showed a significant increase in patients without new AA or ONAA (Table 2).

In the TN cohort, 25% of patients had AA in their BL draw. Similar to the HR+/HER2- cohort, the proportion of patients with new AA decreased with subsequent draws. New AA were found in only 9% of patients with a 2nd draw, all of which occurred in previously unaltered genes. No new AA were detected in the 3rd draw (Table 2 and Fig. 2b). An ONAA was found in 84% of patients in their BL draw, with subsequent draws showing decreasing proportions. The incidence of patients with no new AA or ONAA significantly increased with subsequent draws (Table 2).

In the HER2+ cohort, 38% of patients had AA in their BL draw (not including ERBB2 amplification) and 14–15% of patients had new AA in subsequent draws. All new AA in subsequent draws were new AA in previously unaltered genes (Fig. 2b and Table 2). The incidence of patients with new ONAA and of patients with no new AA or ONAA among the HER2+ cohort are listed in Table 2.

To ensure the validity of our results and mitigate potential selection bias, we performed a separate analysis on a more uniform cohort of patients over time. Specifically, we repeated the analysis only in patients with HR+/HER2- MBC who underwent three or more cfDNA draws (*n* = 79, as shown in Fig. 2c). Even though the proportions were slightly different, the overall trend of decreasing new AA over time was maintained, although AA in previously unaltered genes continued to emerge in serial cfDNA draws.

### New AA in serial cfDNA draws according to gene

**Fig. 3:**
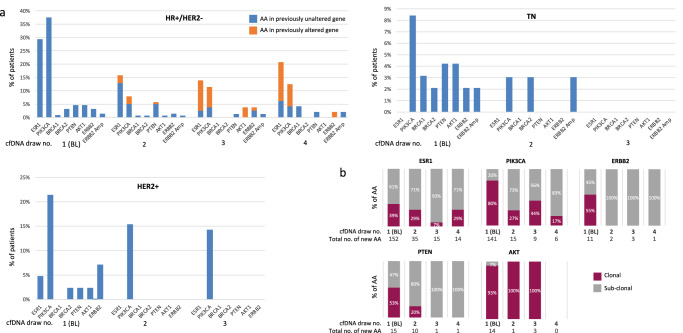
New AA in serial cfDNA analysis according to gene. **a** Incidence of new AA according to gene. **b** Clonality of new AA in serial cfDNA analysis of patients with HR+/HER2- MBC. Clonal, mutation allelic fraction/maximum somatic mutation allelic fraction >50%; Sub-clonal, mutation allelic fraction/maximum somatic mutation allelic fraction < 50%. *In the BL draw all alterations were considered new*.

The frequencies of new AA according to the altered gene in the HR+/HER2- cohort are described in Fig. 3a and Supplementary Table 1. *PIK3CA* and *ESR1* were the most frequent genes with new AA in the HR+/HER2- cohort regardless of draw number. While the majority (82%) of patients with new AA in *ESR1* in the 2nd draw had no previously known *ESR1* actionable mutation, 70% and 82% of the patients with new AA in *ESR1* in the 3rd and the 4th draws, respectively, had new actionable variants in previously altered *ESR1*. Similarly, 33%, 67%, and 67% of new AA in *PIK3CA* were new actionable variants in patients with previously altered *PIK3CA* in the 2nd, 3rd and 4th draws (Fig. 3a and Supplementary Table 1). Moreover, in patients with new AA in previously altered *ESR1* or *PIK3CA* genes in the 3rd or the 4th draws, some of the new actionable variants appeared in the BL or the 2nd draw, disappeared in the 2nd or 3rd draw, and subsequently reappeared in the 3rd or 4th draws (Supplementary Table 1). New AA in *ESR1* were mostly subclonal regardless of draw number. Additionally, while the majority (80%) of *PIK3CA* actionable mutations in the BL test were clonal, the majority of the new *PIK3CA* AA in subsequent draws were subclonal (Fig. 3b). Around 5% of patients had either new *AKT1* or *PTEN* AA in the BL draw. While new alterations in *PTEN* continued to emerge in patients with previously unaltered *PTEN* (although in small numbers), only one patient had a new *AKT1* E17K mutation in the 2nd draw and all patients with new *AKT1* E17K mutations in the 3rd draw had a previous *AKT1* E17K mutation in the BL draw (that was not detected in the 2nd draw and therefore considered as new AA) (Fig. 3a and Supplementary Table 1). *PTEN* AA were mostly clonal (53%) in the BL draw, but mostly subclonal (80–100%) in subsequent draws. In contrast, nearly all new AA (93–100%) in *AKT1* were clonal regardless of the draw number (Fig. 3b). About 4% of patients had AA in *BRCA1* or *BRCA2* in the BL draw. All *BRCA1/2* AA in subsequent draws were loss-of-function mutations in patients with previously unaltered *BRCA*. About 5% of patients had AA in *ERBB2* in the BL draw (3.2% had an actionable mutation in *ERBB2* and 1.5% had new *ERBB2* amplification). Although in small numbers, new *ERBB2* AA continued to emerge in subsequent draws of patients with previously unaltered *ERBB2* (Fig. 3a). All new *ERBB2* actionable mutations in subsequent draws were subclonal (Fig. 3b). Only one patient (1.2%) in the HR+/HER2- cohort had new *NTRK1* AA (*LMNA-NTRK1* fusion) in the 3rd draw.

In the TN cohort, *PIK3CA* was the most frequent gene with AA in the BL draw (8% of patients). Five percent of patients had AA in *BRCA1* or *BRCA2*, and 8% of patients had AA in either *AKT1* or *PTEN*. In addition, 4% of patients had AA in *ERBB2* (mutation or amplification). In the 2nd draw, new AA in *PIK3CA*, *BRCA2*, and *ERBB2* were present at an equal frequency (3% of patients for each). As mentioned above, no new AA were found in the 3rd draw among patients with TNBC (Fig. 3a).

Similar to the other two BC subtypes, in the HER2+ cohort, *PIK3CA* was the most frequently altered gene with AA in the BL draw (21% of patients). AA were also detected in *ESR1* (5%), *BRCA2* (2%) *PTEN* (2%), *AKT1* (2%), and *ERBB2* (7% of patients with an actionable mutation, as *ERBB2* amplifications were excluded for this cohort) in the BL draw, but *PIK3CA* was the only gene with new AA in the 2nd and 3rd draws.

### Correlation between the number and type of prior therapies received and new AA

**Fig. 4:**
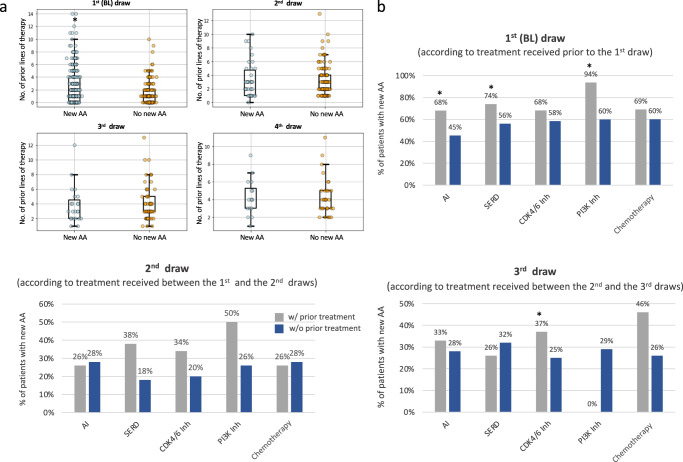
Correlation between number and type of prior treatment received and the incidence of new AA in serial cfDNA analysis of patients with HR+/HER2- MBC. **a** Box plots show number of prior lines of therapy received and the incidence of new AA. Each point represents a patient. The boxes span the inter-quartile range (IQR). Whiskers span 1st quartile −1.5 × IQR and 3rd quartile +1.5 IQR; and points above and below whiskers represent outliers. **b** Type of prior treatments received and the incidence of new AA. *p < 0.05. AI aromatase inhibitor, SERD selective estrogen receptor degrader. *In the BL draw, all alterations were considered new*.

Using logistic regression analysis, we found a significant correlation between the number of prior lines of therapies and the probability of an AA in the BL draw for patients with HR+/HER2- BC (*p* = 0.001). However, no such correlation was found in subsequent draws (Fig. 4a). Figure 4b presents the correlation between type of prior treatment received and the incidence of new AA for patients with HR+/HER2- BC; prior use of AI, SERD or PI3K inhibitor was significantly correlated with the incidence of AA in the BL draw (68% vs. 45% of patients who received vs. did not receive prior AI, respectively; *p* < 0.0001, 74% vs. 56% of patients who received vs. did not receive prior SERD, respectively; *p* = 0.001, and 94% vs. 60% of patients who received vs. did not receive prior PI3K inhibitor, respectively; *p* = 0.002). However, although patients who received prior CDK4/6 inhibitor had a numerically higher incidence of AA in the BL draw compared to patients who did not receive prior CDK4/6, this was not statistically significant (68% vs 58%, respectively; *p* = 0.059). Although some numerical differences were observed, no significant correlation was found between the types of therapy given between the BL and the 2nd draw, and the incidence of new AA in the 2nd draw (Fig. 4b). The use of CDK4/6 inhibitor between the 2nd and the 3rd draw significantly correlated with the incidence of new AA in the 3rd draw (37% vs. 25% of patients who received CDK4/6 inhibitor vs. patients who didn’t receive CDK4/6 inhibitor, respectively; *p* = 0.01)

### Correlation between maximum VAF and incidence of new AA

**Fig. 5:**
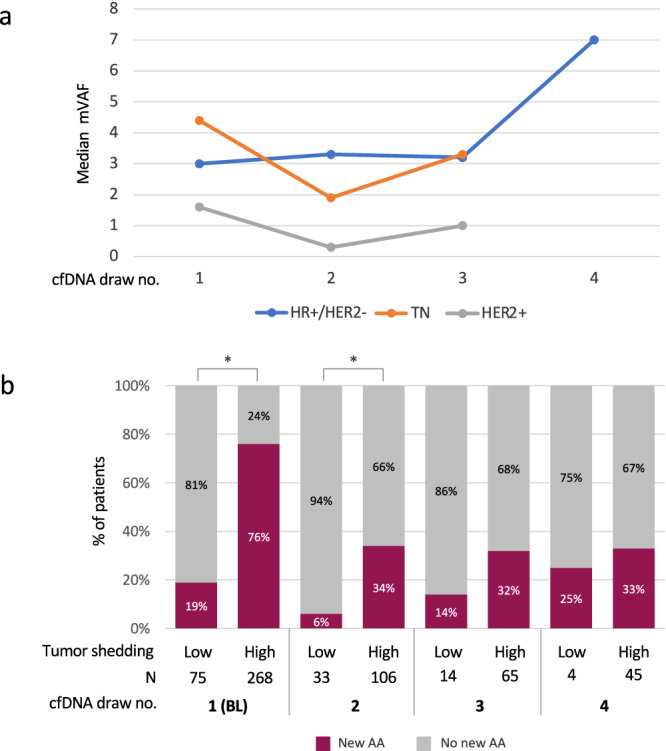
Maximum VAF (mVAF) in serial cfDNA analysis. **a**, Median mVAF trend over time in serial cfDNA analysis across MBC subtypes. **b**, Incidence of new AA according to tumor shedding in patients with HR+/HER2- MBC. Low, mVAF $<$ 0.4; High mVAF $\ge$ 0.4. abbreviations: mVAF, maximum VAF. * *p* < 0.05. *In the BL draw all alterations were considered new*.

To verify that the significant decrease in new AA observed in all cohorts in subsequent cfDNA draws compared to the BL draw (as seen in Fig. 2b and Table 2) was not due to a decrease in overall tumor shedding, we evaluated the trend in median maximum VAF (mVAF) over time. Median mVAF did not significantly vary over time in all three cohorts (Fig. 5a, *p* > 0.05 for all cohorts). The correlation between the level of tumor shedding and the incidence of new AA in the HR+/HER2- cohort is presented in Fig. 5b. Tumors with mVAF < 0.4 were considered as low shedding while those with mVAF > 0.4 were considered as high shedding^14,15^. A significant correlation between the level of shedding and the detection of new AA was found in the BL and the 2nd draws (*p* < 0.0005 and *p* = 0.001, respectively), suggesting a higher probability of detecting a new AA in patients with high shedding tumors. Although the same trend was observed in the 3rd and the 4th draw, it was not statistically significant (Fig. 5b).

### Impact of new AA in serial testing on selection of genotype-matched targeted therapies in HR+/HER2- MBC

An exploratory analysis in the HR+/HER2- MBC cohort was conducted to understand how new AA may impact the selection of genotype-matched targeted therapies. In the HR+HER2- cohort, 24% (84 out of 344) of the patients who underwent a BL draw received matched therapies for the AA identified in that draw, which were administered distal to the draw. Similarly, 7% (10 out of 139) of the patients who had a 2nd draw received matched therapies for new AA (only AA in previously unaltered genes were included) found in the 2nd draw, distal to the draw. Thus, serial testing did inform the selection of additional targeted therapies in a subset of patients. As only 8 patients had new AA in previously unaltered genes in the 3rd or the 4th draw, the sample size was insufficient for conducting the analysis for these cohorts.

## Discussion

Serial cfDNA analysis is increasingly performed for patients with MBC to guide treatment decisions. Nevertheless, the utility of serial cfDNA testing in identifying new AA that may affect treatment selection is not well established. Our study demonstrates that the proportion of patients with MBC identified to have new AA (ESCAT I/II) in serial cfDNA decreased with time, regardless of disease subtype. Nevertheless, new clinically relevant AA continue to emerge, particularly for patients with HR+/HER2- MBC, and did inform the selection of new targeted therapies with serial testing in an exploratory analysis. However, serial cfDNA analysis appeared less effective in providing new clinically meaningful information for patients with TN or HER2 + MBC. For patients with HR+/HER- MBC, some of the new AA in subsequent draws were new actionable variants in the same genes that were known to be altered in previous draws. However, 10-24% of patients had new AA in previously unaltered genes with a potential impact on new treatment selection. The number and type of prior treatments received were found to be correlated with the probability of an AA, mainly in the BL draw of patients with HR+/HER2- MBC.

The overall mutational landscape, encompassing both actionable and non-actionable pathogenic alterations, in our dataset (Supplementary Fig. 2), closely resembled findings from previous blood-based biopsy datasets in metastatic breast cancer (MBC)^16–19^, with *TP53* and *PIK3CA* being the most frequently altered genes in the HR+HER2- and TN cohorts, while *TP53* and *ERBB2* were the most commonly altered genes in the HER2+ cohort. The SAFIR02-BREAST study demonstrated the utility of genotype-matched therapies for patients with MBC harboring ESCAT I/II AA^8^. In our work, 63% of patients in the HR+/HER2- cohort, 25% of patients in the TN cohort, and 38% of patients in the HER2+ cohort had one or more ESCAT I/II AA (AA in *ESR1*, *PIK3CA*, *ERBB2, AKT1, PTEN* or *BRCA1/2*) in their BL draw. Taken together, the high percentage of patients with AA, particularly in the HR+/HER2- cohort, supports the increasingly employed practice of cfDNA testing in the treatment approach for these patients. Importantly, as new targeted therapies for additional genomic alterations are developed and approved, the proportion of patients eligible for these treatments is expected to grow.

Prior studies have evaluated the utility of serial cfDNA collection for patients with MBC in the prediction of clinical outcomes as well as for the detection of newly emerging resistance alterations^20–27^. In our cohort, the total number of alterations detected by serial cfDNA testing increased over time, most likely due to clonal evolution, as also shown by other studies^21,27^. However, the number of new AA or ONAA decreased over time, regardless of subtype (Table 2 and Fig. 2b). In a complementary manner, the proportion of patients with no new AA or ONAA (i.e. those who do not gain any additional clinical benefit from serial testing) increased over time. Interestingly, our results show that patients with HR+/HER2- disease benefit more from serial cfDNA analysis than patients with TN MBC (only 9% of patients with TN MBC had new AA in their 2nd cfDNA draw, and none had new AA in the 3rd draw). Since the total number of alterations in TN MBC tumors is comparable to HR+/HER2- tumors (as shown here and by other studies^17,28^), the lower incidence of new AA in serial cfDNA draws of patients with TN MBC is likely due to prevalent alterations that are not actionable. Moreover, the evidence for actionability of some of the ESCAT I/II alterations, such as *PIK3CA*, is higher for patients with HR+/HER2- compared to the TN subtype^29^.

AA in *ESR1* and *PIK3CA* were the most frequent among patients with HR+/HER2- MBC, regardless of draw number. *ESR1* pathogenic resistance alterations, which promote ligand-independent receptor activation, are rarely detected in the primary tumor and emerge primarily via clonal evolution under the selective pressure of prior endocrine therapy (mainly AI therapy)^28,30–34^. New *ESR1* actionable mutations were found in 29% of patients in the BL draw, 16% of patients in the 2nd draw, and 14% and 21% of patients in the 3 and 4th draws, respectively. However, new *ESR1* AA in patients with previously unaltered *ESR1* were detected in 13%, 3%, and 6% of patients in the 2nd, 3rd, and 4th draws, respectively. Consistent with previous findings^35^, most of the newly detected ESR1 actionable mutations were subclonal (as shown in Fig. 3b), highlighting the potential superiority of cfDNA analysis over a single site tissue biopsy for the detection of newly emerging *ESR1* mutations. Additionally, in our cohort, similar to previous studies^30,34^, prior AI treatment was significantly correlated with the probability of new actionable alterations in the BL cfDNA draw of the HR+/HER2- cohort, probably reflecting emergence of *ESR1* mutations. The oral SERD, elacestrant, that led to improved outcomes for patients with *ESR1-*mutated HR+/HER2- MBC in the recently published EMERALD trial^4^ became an approved genotype-matched therapy for this patient population^36^. Since new subclonal *ESR1* actionable mutations continue to emerge and can be detected by serial cfDNA draws, and a matched targeted therapy is now available, serial cfDNA analysis can potentially be considered in this population^36^.

In contrast to *ESR1* mutations, which are mostly acquired following prior endocrine treatment, *PIK3CA* mutations are found at similar frequencies in primary and metastatic breast tumors, and are generally considered an early event related to tumor initiation^37–40^. In our work, however, 8-12% of patients in the HR+/HER2- cohort developed new AA in *PIK3CA* in subsequent serial cfDNA draws. Moreover, 4–5% of patients developed new AA in previously unaltered *PIK3CA* (Fig. 3a). Our results validate earlier studies demonstrating the emergence of new pathogenic *PIK3CA* alterations in tumors that had previously possessed a wild type (WT) *PIK3CA*, presumably due to disease progression and clonal evolution over time^41,42^. The PI3Kα inhibitor alpelisib in combination with fulvestrant is the standard second-line treatment of *PIK3CA*-mutated HR+/HER2- MBC, based on the SOLAR1 study demonstrating improved outcomes compared to fulvestrant alone^3^. Thus, the detection of newly emerged *PIK3CA* mutation via serial cfDNA analysis in patients without a known *PIK3CA* mutation is of great clinical interest. This work distinguishes between new AA in unaltered genes and new AA in altered genes, as the latter seems to provide less new actionable clinical data. However, Vasan et al. previously showed that tumors harboring several actionable variants in *PIK3CA* are more sensitive to PIK3 inhibition than tumors with a single *PIK3CA* alteration^43^. Therefore, the detection of new actionable *PIK3CA* variants in an already altered gene might also be clinically meaningful.

AKT1 is an additional targetable component of the PIK3/AKT pathway (*AKT1* E17K is considered as ESCAT I AA)^6^. In our cohort of patients with HR+/HER2- cancer, we found new *AKT1* E17K alterations in 5% of patients in the BL cfDNA draw, 1% of patients in the 2nd draw, and 3% of patients in the 3rd draw. Interestingly, all new *AKT1* AA detected in the 3rd draw were found in patients with previously altered *AKT1* gene (specifically, *AKT1* E17K was detected in the BL draw, disappeared in the 2nd draw, and emerged again as a new AA in the 3rd draw) (Fig. 3a). Additionally, as previously described^16,44^, the vast majority of detected *AKT1* E17K alterations were clonal (Fig. 3b). Taken together, our findings suggest the possibility that the AKT E17K mutation, is a dominant alteration that persists throughout tumor evolution.

In our work, 10–24% of patients with HR+/HER2- MBC had newly emerged AA in previously unaltered genes detected by serial cfDNA testing. While our work suggests the potential for serial cfDNA testing to impact clinical decision making, further study is needed to validate this approach, especially as serial cfDNA analyses may not always be covered by private or national insurance, which can create a financial burden for patients and healthcare systems, and not all patients who undergo serial testing may derive clinical benefit.

Limitations of our study include the retrospective nature, which exposed it to biases due to uncontrolled variables. There may be a selection bias for patients who underwent several cfDNA draws throughout their disease course compared to patients who underwent BL draw only. Theoretically, these patients might have had less aggressive disease and a longer disease course, allowing them the time to undergo several cfDNA tests, and these possible differences in tumor biology may affect genomic findings. However, a repeat analysis with a uniform cohort of patients with 3 or more cfDNA draws (Fig. 2c) yielded similar results, reducing the chance of such bias. Selection bias may also occur towards individuals with higher socioeconomic status with commercial insurance that can cover repeated testing. Another limitation is the variation in the timing of cfDNA collection within the draws group. Although for the majority of patients the draws were taken either pre-treatment or during progression, for some patients they were collected while on-treatment. This variation might have led to an underestimation of the number of newly emerged AA, as cfDNA samples collected when a tumor is responding to therapy might have decreased sensitivity and a higher false negative rate due to less tumor shedding in the blood^10^. This could potentially provide an alternative explanation for the observed decrease in new AA during subsequent tests. However, the total number of alterations as well as the mVAF either remained unchanged or significantly increased over time (Table 2 and Fig. 5a, respectively), making this alternative explanation less likely. The variation in the number and type of prior treatments received is also a limitation as distinct treatments may impose selective pressures that can affect the emergence of new AA. Indeed, we found a significant correlation between the number of prior lines of therapies and the probability of AA in the BL draw for patients with HR+/HER2- BC (Fig. 4a). However, no such correlation was found in subsequent draws, perhaps due to the smaller sample sizes. Additionally, some of the sample sizes of the different draw groups (mainly subsequent draws in the TN and HER2+ cohort) were relatively small, which might have affected the statistical power of the analysis. Finally, a major limitation is the use of the ESCAT scale as a framework for determining actionability, due to the constantly evolving spectrum of approved genotype-matched therapies, making this type of analysis valid mainly for the time period during which it was conducted. Indeed, while an exploratory analysis of HR+/HER2- MBC here did demonstrate that serial testing led to the selection of new targeted therapies in a subset of patients, this analysis was limited by the retrospective nature of this study and evolving drug approvals during the study time period (for example, alpelisib was not approved until midway through the study period, and elacestrant was not approved in the study period, which impacted our analyses). Additionally, for later test serial results, the sample size was not sufficient for conducting the analysis, and thus, this approach needs to be study further. However, while the limitations of the study arise from its retrospective nature and real-world patient population, using real-world data is also a strength as it reflects the diversity of patients and clinical situations, and can be used to inform clinical decisions in real-world settings. Finally, this study is constrained by the inherent challenges associated with liquid biopsy, which encompass uncontrollable biological factors affecting cfDNA, potential sample collection and handling issues and variable tumor-derived cfDNA quantities potentially impacting reproducibility and sensitivity. Furthermore, NGS read errors can result in false-positive variant calls while inadequate representation of the original cfDNA molecules by NGS may lead to false negatives^45^. Moreover, the utilization of commercial NGS panels (such as the Guardant360® employed in this research) imposes limitations in the detection of structural genomic rearrangements^46^ and offers a restricted genotyping panel, in contrast to whole exome sequencing.

In conclusion, our study evaluates the spectrum of ESCAT I/II AA detected by serial cfDNA analysis in patients with MBC. Despite a decrease in the incidence of new AA with subsequent draws, some new clinically relevant targetable alterations continue to emerge, particularly for patients with HR+/HER2- MBC, on serial testing. These findings suggest the potential for using serial cfDNA testing throughout the disease course of this patient population to identify actionable mutations with clinical relevance. However, further research is needed to validate these findings in a larger cohort, determine the optimal timing of serial cfDNA testing, identify patient subgroups who would benefit most from this approach, and evaluate the impact of serial cfDNA analysis on the selection of genotype-matched therapy and patient outcomes. Additionally, Future analyses may also consider evaluating the emergence of new actionable alterations in serial biopsies of metastatic lesions after receipt of varying therapies. The cost-effectiveness of serial cfDNA in impacting clinical decision making also needs to be ascertained and requires further research.

## Methods

This study was performed in compliance with the Declaration of Helsinki. The retrospective analyses were performed with IRB approval from an institutional protocol (Massachusetts General Hospital). Per IRB regulations, individual patient consent was not required for this retrospective analysis, although all patients had been consented for Guardant360® cfDNA testing prior to collection.

### Study population

Consecutive patients with MBC who underwent plasma-based cfDNA testing (Guardant360®, 74-gene assay) as part of routine clinical care at Massachusetts General Hospital at any time after their diagnosis of MBC between January 2016 and June 2021 were identified. The test was offered for patients with MBC seen at the center during this period and was conducted for patients who consented for testing. All patients with known MBC subtype and at least one cfDNA test result available for analysis were included. Tumor subtype was determined from pathology reports of the metastatic specimen, or if unavailable, the primary tumor. MBC subtype was designated HR+/HER2-; estrogen receptor (ER) and/or progsterone receptor (PR) > 1% and HER2 IHC 0/1+ or 2+ and FISH non-amplified), HER2 positive (HER2+; HER2 IHC 3+ or 2+ and amplified FISH) or triple-negative (TN; ER < 1%, PR < 1% and HER2 negative). Patients were divided into 3 cohorts (HR+/HER2-, TN, HER2+) based on their MBC subtype (Fig. 1a). The first cfDNA draw for each patient was identified and defined as the baseline (BL) draw. For the group of patients with subsequent serial draws, each subsequent draw was serially defined as the 2nd, 3rd or 4th cfDNA draw according to the order in which these draws occurred. Due to very small numbers of patients who underwent a 4th draw in the TN and HER2+ cohorts, the 4th draw was included only for patients in the HR+/HER2- cohort. For patients with >4 draws in the HR+/HER2- cohort or >3 draws in the TN and the HER2+ cohorts, additional draws were excluded. A retrospective review of medical records (Institutional Review Board-approved institutional protocol) was performed to identify patient demographics, treatments received before and after each cfDNA draw, time interval between draws, and cfDNA genomic results. The type/timing of draw was determined as follows: *pre-treatment*, if no therapies for metastatic disease were received prior to cfDNA draw (draw done at time of MBC diagnosis) or if cfDNA draw occurred <30 days after starting 1st line of therapy for MBC; *on-treatment*, if cfDNA draw occurred >30 days after starting therapy and <30 days before completing therapy; *post-treatment discontinuation* if cfDNA draw occurred <30 days before ending therapy and <30 days after starting the next therapy. The vast majority of post-treatment discontinuation draws occurred at time of disease progression.

### cfDNA sequencing and new alterations analysis

cfDNA sequencing was performed as part of routine care, using the commercially available Guardant360^®^ sequencing platform (74 cancer-related genes) as previously described^47,48^. The steps taken to analyze cfDNA results are presented in Fig. 1b. For patients with serial draws, new cfDNA alterations in each draw, compared to the previous draw (2nd vs. 1st, 3rd vs. 2nd, 4th vs. 3rd), were quantified and characterized. The pathogenicity of new alterations was determined using the OncoKB precision oncology database (available from: https://www.oncokb.org/)^49^. New pathogenic alterations were further classified as actionable alterations (AA) or oncogenic non actionable alterations (ONAA) using the ESCAT scale^5,6^. Alterations that met the ESCAT I (alteration-drug match was associated with improved outcomes in clinical trials) or ESCAT II (alteration-drug match was associated with antitumor activity) criteria were considered AA. A list of ESCAT I/II alterations is included in Fig. 1b. AA in *ESR1, PIK3CA*, *ERBB2* (mutation or amplification), *AKT1*, *PTEN*, *BRCA1/2*, and *NTRK* were included while data on MSI-H and TMB-H alterations was not available for this analysis. Pathogenic alterations that did not meet ESCAT I/II criteria were classified as ONAA. By comparing the new AA in subsequent draws to the patient’s cfDNA results from all previous draws (2nd vs. 1st, 3rd vs. 1st and 2nd, 4th vs. 1st, 2nd and 3rd), new AA were further classified as either ‘new AA in previously unaltered gene’ or ‘new AA in previously altered gene,’ with the latter referring to a new actionable variant in a previously altered gene (for example, new actionable variant in *ESR1* for a patient with a previously detected AA in *ESR1*, as demonstrated in Fig. 1b). A new actionable variant in a previously altered gene that was detected in a subsequent draw was defined as a ‘new AA in previously altered gene’, regardless of whether the old actionable variant continued to be detected in the subsequent draw. Both somatic and germline AA were included in the analysis.

### Tumor shedding and clonality analysis

The variant allele fraction (VAF), which was provided for each mutation in the Guardant360® analytic report, was calculated as the number of mutated DNA molecules divided by the total number of DNA fragments at that allele (mutated plus wild type). The highest somatic VAF was determined as the maximum VAF (mVAF) for each draw, regardless of the gene correlated to this VAF^50,51^. mVAF was used as a surrogate for tumor shedding. Because germline alterations’ VAFs do not correlate with tumor shedding, germline alterations were excluded from this specific analysis based on VAF frequency around 50% +/−5%, type of alteration and VAF of other alterations in the same test. Based on the median level of detection of the sequencing platform, cfDNA draws with a mVAF $\ge$ 0.4 were considered to represent high-shedding tumors, while those with a mVAF <0.4 were considered to represent low-shedding tumors^14,15^. The VAF/mVAF calculation was used to determine clonality for actionable mutations. An actionable mutation was considered *clonal* when VAF/mVAF $\ge$ 50% and was considered *subclonal* when VAF/mVAF < 50%^16,35^.

### Genotype-matched targeted therapies analysis

3Patients in the HR+/HER2- cohort who received matched therapies for new AA found in the BL or the 2nd draw, after the draw, were identified. Supplementary Table 2 summarizes the AA and corresponding related genotype-matched therapies. For the 2nd draw, only new AA in previously unaltered genes were included in the analysis

### Statistical analysis

The proportions of patients with new AA and ONAA were compared between subsequent draws and the BL draw, using a two-tailed test of proportions. Median number of alterations as well as median mVAFs were compared between subsequent draws and the BL draw using the Wilcoxon rank-sum test. The association between the number and type of prior lines of therapy and the probability of new AA was analyzed using logistic regression analysis. The correlation between the level of tumor shedding and the probability of new AA was analyzed using the two-sample Wilcoxon rank-sum test. Kruskal–Wallis *H* test was used to compare median mVAF of draws over time. Statistical analysis was performed using Stata (StataCorp. 2021. Stata Statistical Software: Release 17. College Station, TX: StataCorp LLC.).

### Supplementary information

Supplemental Material

Reporting checklist

## Data Availability

Data used in this study is not publicly available as it contains patient information including genomic/genetic information, which cannot be released as this is not covered by the informed consent signed by patients. Clarifications on the study data may be requested in writing to the corresponding author.
