# Supplementary material for: Genomic spectrum of actionable alterations in serial cell free DNA (cfDNA) analysis of patients with metastatic breast cancer
Source: NPJ Breast Cancer. 2024 Apr 11;10:27. doi: 10.1038/s41523-024-00633-7 (PMC11009384; doi:10.1038/s41523-024-00633-7)

Supplementary Table 1 – Patients with new AA (ESCAT I/II) in serial cfDNA analysis according to the prior status of the altered gene in the HR+/HER2- cohort.
** In the BL draw all alterations were considered new.*
*** New actionable variants that were detected in the BL/1st draw, disappeared in the 2nd/3rd draw and subsequently reappeared in the 3rd or 4th draws.*
*** The bottom row (any AA) is not a sum of the above rows as more than one AA could appear in the same tumor.

	1st (BL) draw * N=344		2nd draw N=139		3rd draw N=79			4th draw N=49		
**Patients with new AA , n (%)**						**Previously altered genes**			**Previously altered genes**	
	**previously unaltered genes**	**previously altered genes**	**previously unaltered genes**	**previously altered genes**	**previously unaltered genes**	New actionable variants were never detected	New actionable variants were previously detected**	**previously unaltered genes**	New actionable variants were never detected	New actionable variants were previously detected**
ESR1	101 (29)	N/A	18 (13)	4 (3)	2 (3)	5 (6)	4 (5)	3 (6)	2 (4)	5 (10)
PIK3CA	129 (38)	N/A	7 (5)	4 (3)	3 (4)	1 (1)	5 (6)	2 (4)	2 (4)	2 (4)
BRCA1	3 (1)	N/A	1 (1)	0 (0)	0 (0)	0 (0)	0 (0)	2 (4)	0 (0)	0 (0)
BRCA2	11 (3)	N/A	1 (1)	0 (0)	0 (0)	0 (0)	0 (0)	0 (0)	0 (0)	0 (0)
PTEN	16 (5)	N/A	7 (5)	1 (1)	1 (1)	0 (0)	0 (0)	1 (2)	0 (0)	0 (0)
AKT1	16 (5)	N/A	1 (1)	0 (0)	0 (0)	0 (0)	3 (4)	0 (0)	0 (0)	0 (0)
ERBB2	11 (3)	N/A	2 (1)	0 (0)	2 (3)	1 (1)	0 (0)	0 (0)	0 (0)	1 (2)
ERBB2 amplification	5 (1)	N/A	1 (1)	0 (0)	1 (1)	0 (0)	0 (0)	1 (2)	0 (0)	0 (0)
**Any AA*****	**217 (63)**	**N/A**	**33 (24)**	**5 (4)**	**8 (10)**	**15 (19)**		**8 (16)**	**8 (16)**	

Supplementary Table 2 – AA and corresponding related genotype-matched targeted therapies.
*SERD is selective estrogen receptor degrader.

Altered Gene	Matched therapy
PIK3CA	PI3K inhibitor (Alpelisib or investigational)
ESR1	SERD*/oral SERD (Fulvestrant, Elacestrant or investigational)
ERBB2 mutation/ *ERBB2* amplification	Anti-HER2 directed therapy (Trastuzumab, Pertuzumab, Trastuzumab emtansine (T-DM1), Neratinib, Lapatinib, Tucatinib, Trastuzumab deruxtecan (T-DXd) or investigational)
BRCA1/BRCA2	PARP inhibitor (Olaparib, Talazoparib, or investigational)
PTEN/AKT1	AKT inhibitor (investigational)

Supplementary Figure 1. **Trends in the number of new alterations between the baseline (BL) and subsequent draws at the patient level**. **a**, HR+/HER2- cohort. **b**, TN cohort. **c**, HER2+ cohort. For each patient, the number of new alterations in the BL draw was set as the reference point at 100%, and the number of new alterations in subsequent draws is presented as a percentage relative to the BL. The red dashed line within the boxes represents the median number of new alterations for each draw. The blue dots indicate the number of new alterations per patient, and the lines connecting the dots illustrate the changes across the draws for each patient.

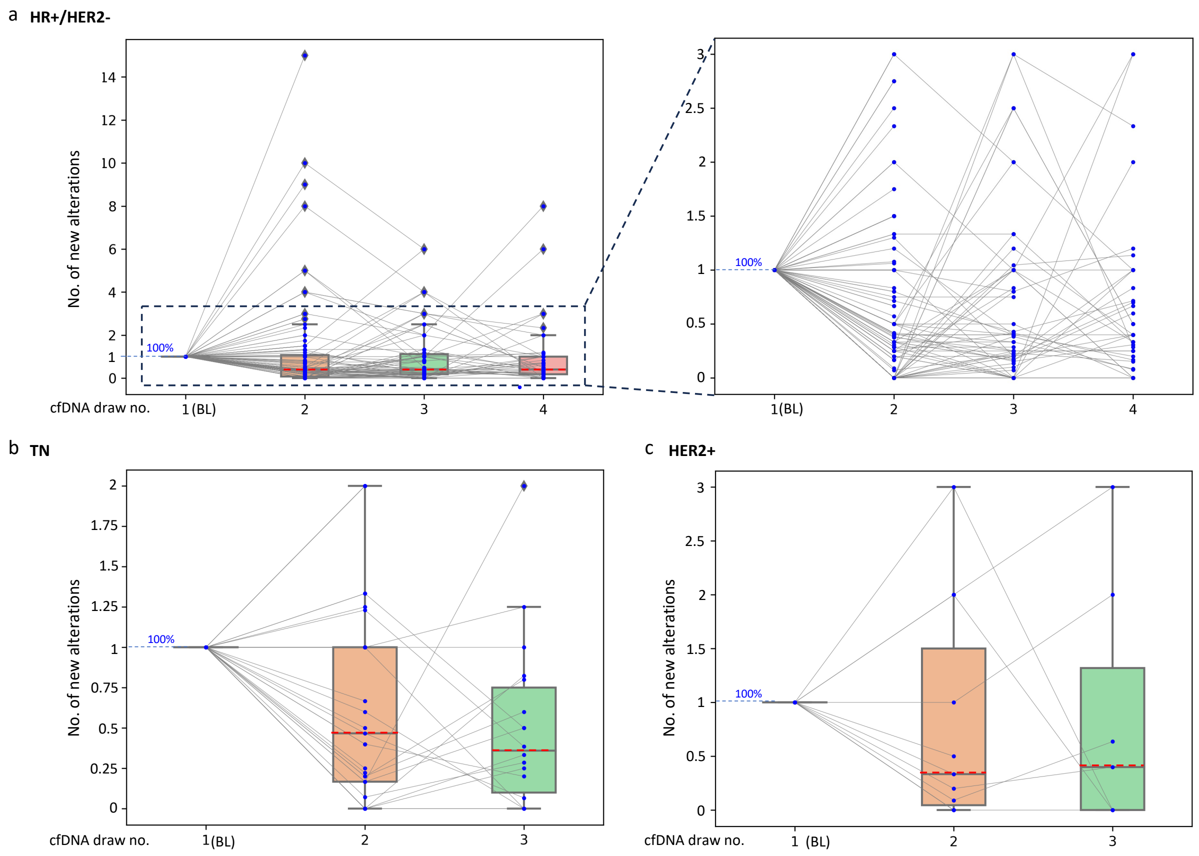

Supplementary Figure 2. **Genomic landscape of pathogenic alterations (actionable and non-actionable) in the BL draw according to breast cancer subtype. a**, HR+/HER2- cohort. **b**, TN cohort. **c**, HER2+ cohort. Only oncogenic/likely oncogenic alterations are presented (see Methods). Cases in which more than 2 pathogenic alterations were detected in the same gene for the same patient are represented as Multiple (dark brown boxes).

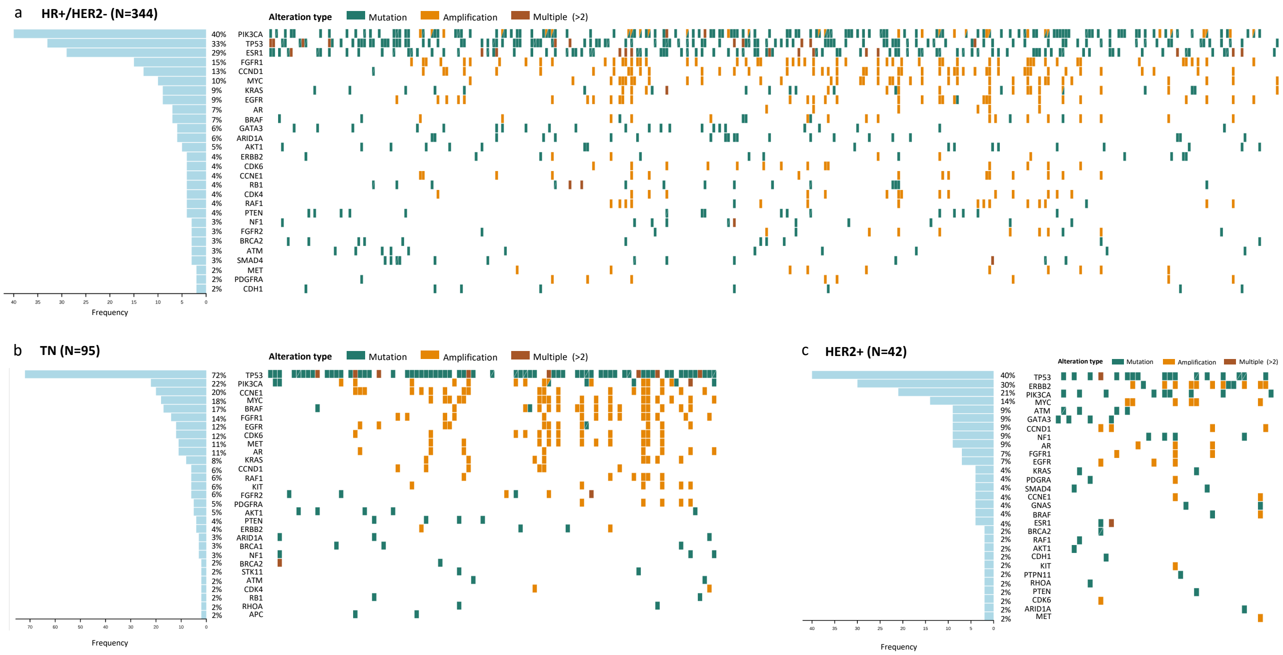

Supplement: Supplementary file 1 — Supplemental Material [file 41523_2024_633_MOESM1_ESM.pdf]
